# Protective Factors in the Use of Electronic Media According to Youth and Their Parents: An Exploratory Study

**DOI:** 10.3390/ijerph18073573

**Published:** 2021-03-30

**Authors:** Luísa Campos, Lurdes Veríssimo, Bárbara Nobre, Catarina Morais, Pedro Dias

**Affiliations:** 1 Faculty of Education and Psychology, Universidade Católica Portuguesa, Rua Diogo Botelho, 1327, 4169-005 Porto, Portugal; lverissimo@porto.ucp.pt (L.V.); barbaranobre12@gmail.com (B.N.); cmorais@porto.ucp.pt (C.M.); pdias@porto.ucp.pt (P.D.); 2 Research Centre for Human Development, Rua Diogo Botelho, 1327, 4169-005 Porto, Portugal

**Keywords:** youth, electronic media, extracurricular activities, parental mediation, protective factors

## Abstract

The use of electronic media (EM) by youths has been widely described in the literature, indicating the relevance of understanding the factors that can protect against its risks. We aimed to explore the protective role of participating in extracurricular activities (ECAs) and of parental mediation in the use of EM by young people. A total of 1413 people (729 students, aged between 11 and 17 years old, and one of their parents) participated in this study. Youths who engaged in ECAs spent significantly less time per week on EM and perceived that the use of EM devices had less of a negative impact. When parents and their children presented a congruent notion of how much time youth spent on EM, parents perceived EM to have less of a negative impact on their children compared to dyads with discrepant assessments. The hierarchical regression results indicated that regardless of time spent per week on EM, engaging in ECAs was a significant predictor of perceiving a less negative impact, playing a role as a protective factor regarding the use of EM. The ubiquity of EM reinforces the importance of the focus of this study, and its results contribute to creating specific guidelines for parental education and educational policies.

## 1. Introduction

Electronic media (EM) are part of people’s daily lives, being perceived as a relevant resource for learning, communication, and entertainment [1,2,3,4,5]. Despite this, some challenges have been highlighted [3,6,7], especially concerning the prominent and significant interaction of young people with EM [1,2,5,8], since this occurs during a developmental period involving intense psychological transformations (e.g., identity exploration, self-expression, and peer acceptance) [5,9,10]. EM use tends to increase with age. The EU Kids Online 2020 report [11] displayed that youths of 14–16 years old spend almost twice as much time online as 9–10-year-olds, and also use a broader range of online activities.

Some research suggests that young people who spend more time on EM report lower socioemotional skills (e.g., self-management, motivation, and responsible decision-making) [12] and poor psychological adjustment [13]. Additionally, young people who use EM are more likely to perpetrate or become victims of bullying [12] and cyberbullying [14,15,16], and to display risky behaviors (e.g., contact with strangers, pornography, and sexual messages) [7,16,17]. Regarding interpersonal relationships, the literature is not univocal: some researchers emphasize the relationship between greater use of technologies and lower real communication skills [5]; others highlight its importance as a significant context for socialization and interaction [18,19].

In summary, over the past few decades, research has focused on the negative impact of EM use on children’s and adolescents’ well-being [5,16,20], while being progressively concerned about its opportunities [3,4,18]. Nowadays, it is crucial to identify and promote protective factors for these negative psychological, social, and behavioral outcomes, even more so in the current context arising from the COVID-19 pandemic, since young people are inevitably more connected to EM [21]. Therefore, the developmental environments, such as family and extracurricular activities (ECAs), may play an important role as protective factors, allowing the balance of virtual with real interactions and promoting socioemotional development.

ECAs have been defined as structured activities occurring outside of school hours, involving adult supervision, and including an overall goal of promoting positive development [22,23]. More specifically, beyond the opportunities to develop technical knowledge and skills, receive adult mentoring, and build new relationships, ECAs have been considered an ecological context that contributes to youths’ psychological, social, and emotional wellbeing [22,24,25,26]. Fredricks and Eccles [27] found a positive relationship between participation in ECAs and better academic adjustment, psychological competencies, and a positive peer context. More recently, this relationship has been hypothesized as a result of a transfer of non-cognitive skills between ECAs and academic activities [28]. In sum, ECAs provide structured time, with specific tasks that demand the use of a set of socioemotional skills in real time and with others, as opposed to tasks that require media use (e.g., videogames), which often do not receive adult supervision, and can offer an escape of not dealing with negative emotions (e.g., if losing, participants can easily turn off the device, not dealing with frustration), not developing the necessary socioemotional skills. Age differences concerning youths’ involvement in ECAs tend to exist according to changes regarding the developmental stage and the type of activities performed. Participation in ECAs seems to increase between middle childhood and early adolescence [22]. In this transition, youths shift from a stage of exploring a wide range of activities to more stable interests or commitments, deciding to focus on a particular or a limited number of activities [22,29]. Furthermore, over the course of adolescence, ECAs tend to have a more significant role and salience in youths’ lives and offer additional developmental opportunities (e.g., assume leadership roles, solve problems, and build solutions) [27].

Similarly, studies have explored the role of the family as an influential social environment that can shape youths’ EM experiences [30,31,32]. Parental mediation consists of “the diverse practices through which parents try to manage and regulate their children’s experiences with the media” [33] (p. 7). Past research has been particularly interested in the parental mediation of children’s use of television [34]. Over the last few decades, given the technological development, the focus has shifted to other media devices (e.g., computers, tablets, smartphones) and their use (e.g., internet access, social media). One of the most recent approaches to media use parental mediation, developed by Livingstone [33], describes it via five different strategies: (1) active mediation of internet use or co-use, which encompasses an active sharing of the youth’s online experiences, such as parents sitting nearby whilst the child is online; (2) active mediation of internet safety, in which parents’ actions are more focused on promoting safe and responsible uses of the internet; (3) restrictive mediation refers to practices imposed by parents to restrict youth’s use, such as limiting their time spent online, and where, when and how children can use it; (4) technical restrictions refer to the use of software and/or other technical tools to monitor or restrict children’s online activities; and (5) monitoring, which includes an active check from parents of the online contents after children’s use. 

According to the parental mediation theory [34], these mediation strategies used by parents can mitigate the negative effects that media use have on their children. Bleakley et al. [32] suggest that parental involvement and a better quality of family relationships seem to be associated with less problematic use of the internet. The concept of active mediation and co-use has been reported as more effective in reducing the risks associated with EM [30]. Family environments of trust and closeness may promote young people to report situations that bother them [5,16], since young people can be considered the best source of information about the activities they perform on EM [35].

Despite the relevance of identifying and promoting protective factors of EM use, these still need to be explored. Thus, we aimed to examine the protective role of participation in ECAs and parental mediation in the use of EM by young people. Moreover, research is still scarce, especially that which considers the perspectives of both the youth and their parents. Congruence, and particularly incongruence, between parent’s and youth’s perceptions is an important issue to attend to, as it can reflect important information regarding family’s cohesion and/or organization [36]. Specifically, this (in)congruence may help to explain difficulties related to their relationship, such as conflict and communication issues [37,38]. Therefore, the specific goals of the study were (1) to characterize and compare time spent on EM and the perceived negative impact of EM assessed by youth and parents; (2) to examine the relation between youth’s age, time spent on ECAs and on EM, and perceived negative impact of EM; (3) to examine group differences in the amount of time spent by youths on EM and on the perceived negative impact of EM, considering those who engage and who do not engage in ECAs, and those reporting parental mediation in EM versus those who do not report such mediation; and (4) to analyze the predictive power of ECA engagement and parental mediation as protective factors against the negative impacts of EM use.

## 2. Materials and Methods

### 2.1. Participants

The sample included both youths and one of their parents. Regarding youth participation, 729 young students (57% girls), aged between 11 and 17 years old (M = 13.46, SD = 1.03), agreed to participate in this study. They were all in 7th to 9th grade, from both private and public schools and from all regions of Portugal. Table 1 summarizes the participants’ sociodemographic characteristics. The majority of the youth lived with both parents (76%), whereas the remaining participants lived with one biological parent. The parent sample was composed of 684 participants, who were mostly mothers (75%) and currently employed (84%; 14% unemployed and 2% retired).

### 2.2. Measures

#### 2.2.1. Media Activity Form (MAF)—Youth Self-Report; Media Activity Form—Parent Report 

The media activity form (MAF)—youth self-report and parent report [39] was used for collecting data regarding the use, and the perceived impact of the use, of media in youth based on their self-reports and on parents’ reports. The MAF includes three sections. The first section aims to collect basic socio-demographic data (sex, age, and school year). The second section focuses on media activity use. It is composed of 13 items concerning different activities within media (e.g., social networking on Facebook, Instagram, etc.). In this section, participants state how long (in hours and minutes) they spend, or their children spend, on each of the activities on a typical weekday, on a typical Saturday, and on a typical Sunday. The third section examines the youths’ or parents’ perception of the negative impact of media activity. It includes 11 items (e.g., “I would rather be on media than do things with my family” for the youth self-report; “My child would feel better if they spent less time on media” for the parent report) with 3 possible responses based on a Likert scale: participants score 0 if the statement is not true; 1 if the statement is somewhat or sometimes true; 2 if the statement is very true or often true. 

The MAF was translated to Portuguese as part of a larger international project. The translation process included a think-aloud procedure and a back-translation conducted by a North American native English-speaker. The translated version was approved by the author. The items concerning the negative impact of media use showed good reliability in the present study’s sample (Cronbach’s α = 0.81 for the youth self-report and 0.87 for the parent report).

#### 2.2.2. Media Use Parental Mediation and Extracurricular Activities Form

The media use parental mediation and extracurricular activities form is a section of a larger characterization questionnaire developed within the *Media Activity and Mental Health Project,* in Portugal. This section includes one question related to the existence of rules defined by parents concerning the use of EM (“Do your parents define any rules related to the use of electronic media devices?”, answered in a dichotomous way (“Yes/No”)). This form also includes four questions concerning participants’ involvement in ECAs (“Are you involved in any extracurricular activity [e.g., Ballet, Football, Scouts]?”, answered in a dichotomous way [“Yes/No”]), followed by a description of the ECA, its weekly frequency, and its duration.

### 2.3. Data Collection and Analysis Procedures

Data were collected in February and March 2019 by the research team in 17 public and private schools across the entire country. Authorization from the schools’ boards of directors and from the Ministry of Education was obtained (registration no. 0128800006), in accordance with all the ethical requirements for research. Informed consent was obtained from participants and their parents. At the time of data collection, each student received an envelope with a sheet explaining the study and the research protocol. Anonymity and confidentiality were assured using a pre-established code, allowing for pairing parents’ and youths’ answers.

The analysis was performed using the statistical analysis program IBM SPSS Statistics^®^ v.26.0 (IBM Inc., Armonk, NY, USA). Before testing the hypotheses, normality assumptions were checked. The amount of time per week spent on EM did not meet this assumption (skew (sk) = 3.89, kurtosis (ku) = 28.67; [40]). Thus, two young participants were removed from the analysis including this variable as they were identified as outliers (spending over 85 h/week). Without these two participants, the data did not present severe deviations from normal distribution in any of the variables of the study (|0.30| *> sk* < |1.92|, |0.28| > *ku <* |5.06| [40]).

## 3. Results

### 3.1. Time Spent on ECAs and EM Assessed by Youth and Parents

A total of 531 youth (73%) were engaged in some kind of ECA (e.g., dance, basketball, scouts, football, learning a language, swimming). Of these, 364 (69%) engaged in a sport ECA. The time they spent on their main ECA varied from one to seven times a week, for a total of 30 min to 7 h a week (M = 97 min, SD = 43.80 min). Regarding the use of EM, 98% of the youths reported using a smartphone, 89% a computer, 62% a tablet or PC, and 21% other devices.

The MAF instrument allowed us to collect data regarding the amount of time children spent on the different EM on a typical day of the week, on a typical Saturday, and on a typical Sunday. The average of time spent on each device per day was multiplied by the 5 days of the week, and then added to the average of time spent on a typical Saturday and Sunday. The same procedure was used to calculate parents’ perceptions regarding the amount of time their children spent per week on EM.

The youths reported spending, on average, 9 h 31 min on EM (SD = 6 h 38 min) in a typical week, and most of them (71%) had some kind of parental mediation in the use of these devices. The perceived negative impact of EM reported by youth was, on average, 0.69 (SD = 0.39). Parents reported that their children spend, on average, 8 h 59 min (SD = 7 h 28 min) on EM in a typical week. The perceived negative impact of EM reported by parents was, on average, 0.72 (SD = 0.48).

### 3.2. Comparison of Youths’ vs. Parents’ Perceptions of Time Spent on EM and Perceived Negative Impact of EM

Paired-sample *t*-tests were conducted to test the differences among youths’ and parents’ perceptions; thus, only the completed dyads (i.e., when both child and one parent completed the survey) were used. Overall, parents perceived their children to spend significantly less time on EM (M = 8 h 45 min, SD = 6 h 20 min) compared to the amount of time reported by the youths (M = 9 h 15 min, SD = 6 h 52 min) (*t*(618) = 34.39, *p* < 0.001, *g* = 0.11). However, no differences were found regarding the perceived negative impact of these devices (parents: M = 0.72, SD = 0.48, children: M = 0.69, SD = 0.39, *t*(700) = 1.70, *p* = 0.089).

Children and parents differed in their reported time spent on EM, on average, by 3 h 31 min (SD = 4 h 50 min), ranging from 0 (perfect consonance) to 36 h 42 min. Percentiles (33%) were used to categorize the congruency between how much time parents perceived children to use these devices for, and how much time children reported actually using them, and a one-way ANOVA was used to test the differences regarding the perceived negative impact of EM amongst the three groups (Table 2).

When congruency was higher, parents perceived EM to have less of a negative impact on their children (M = 0.59, SD = 0.48) compared to the groups showing a discrepancy (parents > children: M = 0.76, SD = 0.48, *p* = 0.001; children > parents: M = 0.75, SD = 0.47, *p* = 0.002) (*F* (2,606) = 8.23, *p* < 0.001). There were no differences amongst both discrepancy groups (*p* = 0.801). The same pattern arose when we analyzed the perceived negative impact reported by children: when congruency was higher, children perceived less of a negative impact *(M = 0.58, SD* = 0.39) compared to the discrepancy groups (parents > children: M = 0.70, SD = 0.37, *p* = 0.002; children > parents: M = 0.74, SD = 0.38, *p* < 0.001) (*F* (2,606) = 8.23, *p* < 0.001). There were no differences amongst the discrepancy groups (*p* = 0.302).

### 3.3. Relation between Age, Time Spent on ECAs, Time Spent on EM, and Perceived Negative Impact of EM

Table 3 shows the Pearson correlation coefficients among the different variables of the study. The results showed a positive association of age with time spent on EM and perceived negative impact of EM, reported by both youth and parents. Time spent on EM was also positively correlated with both youths’ and parents’ perceived negative impact of EM. Time spent on ECAs was negatively associated with the perceived negative impact of EM, assessed by both respondents. No association was found between time spent on EM and time spent on ECAs.

### 3.4. Group Differences in the Amount of Time Spent by Youth on EM and in the Perceived Negative Impact of EM

#### 3.4.1. Youths’ Perceptions

Independent-sample *t*-tests were conducted to test the differences among youths who engaged (vs. did not engage) in ECAs.

Regarding the engagement in ECAs, the results showed that youth who did not engage in any activity spent significantly more time per week on EM (M = 11 h 31 min, SD = 7 h 40 min), and perceived a more negative impact deriving from the use of these devices (M = 0.77, SD = 0.43), than youth who engaged in some kind of ECA (time spent on EM: M = 8 h 50 min, SD = 6 h 6 min; *t*(246) = 4.13, *p* < 0.001, *g* = 0.44; perceived negative impact: M = 0.66, SD = 0.38, *t*(303) = 3.13, *p* = 0.002, *g* = 0.28).

An independent-sample *t*-test was also conducted to compare the time spent per week on EM reported by children with and without parental mediation. Children whose parents enforced restrictions on the use of EM reported spending less time on these devices (M = 9 h 4 min, SD = 6 h 21 min) compared to those whose parents did not impose any kind of restrictions (M = 10 h 46 min, SD = 7 h 12 min; *t*(270) = 2.74, *p* = 0.006, *g* = 0.22), but these groups did not differ in their perceptions of the negative impact of EM (*t*(696) = 0.43, *p* = 0.671).

#### 3.4.2. Parents’ Perceptions

The results from independent-sample *t*-tests show that parents who defined rules related to their children’s use of EM perceived their children to spend less time per week on these devices (M = 8 h 37 min, SD = 7 h 33 min) than those who did not engage in this kind of parental practice (M = 9 h 57 min, SD = 7 h 16 min) (*t*(631) = 1.96, *p* = 0.050, *g* = 0.16).

There were no differences regarding the perceived negative impact of these devices on their children among parents who defined rules (M = 0.71, SD = 0.50) compared to those who did not (M = 0.72, SD = 0.47) (*t*(695) = 0.14, *p* = 0.887). However, parents whose children engaged in ECAs perceived these devices to have less of a negative impact (M = 0.70, SD = 0.48) compared to parents whose children did not engage in ECAs (M = 0.77, SD = 0.49) (*t*(706) = 1.97, *p* = 0.049, *g* = 0.14).

### 3.5. Predictors of Perceived Negative Impact of EM

#### 3.5.1. Youth’s Perceptions

**Figure 1:**
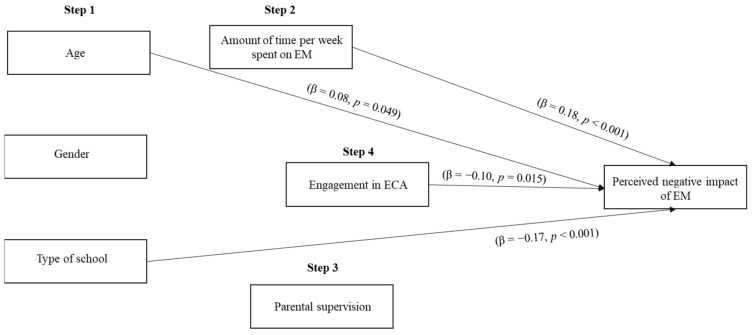
Predictors of perceived negative impact of EM (youth’s sample).

A hierarchical regression was conducted to analyze the predictive power of ECA engagement and parental mediation on the negative impact of EM use (see Figure 1). In step 1, age, sex (1 = male, 2 = female), and type of school (1 = public, 2 = private) were included. The amount of time per week spent using EM was included in step 2; the existence of rules defined by parents regarding the use of EM (0 = no rules, 1 = rules) was included in step 3; and whether they engaged in ECAs (0 = no, 1 = yes) was included in step 4. The regression model was significant (*F*(6,558) = 12.55, *R*^2^ = 0.12, *p* < 0.001). The results showed that young participants’ age (*b* = 0.03, β = 0.08, *t* = 1.97, *p* = 0.049), type of school (*b* = −0.14, β = −0.17, *t* = −4.08, *p* < 0.001), and amount of time per week spent on EM (*b* = 0.01, β = 0.18, *t* = 4.12, *p* < 0.001) were significant predictors of the perceived negative impact of EM. Thus, those studying in a public school perceived a more negative impact of the use of these devices. The older the students and the more time per week spent on EM, the more negative the perceived impact of EM. Their sex (*p* = 0.261) and the existence of rules (*p* = 0.083) did not predict the perceived negative impact of EM. More importantly, regardless of their age, sex, type of school, the existence of rules, or the time they spent on EM, their engagement in ECAs was a significant negative predictor of the perceived negative impact of EM (*b* = −0.09, β = −0.10, *t* = −2.43, *p* = 0.015), meaning that participants who engaged in these activities perceived less of a negative impact of these devices.

#### 3.5.2. Parents’ Perceptions

**Figure 2:**
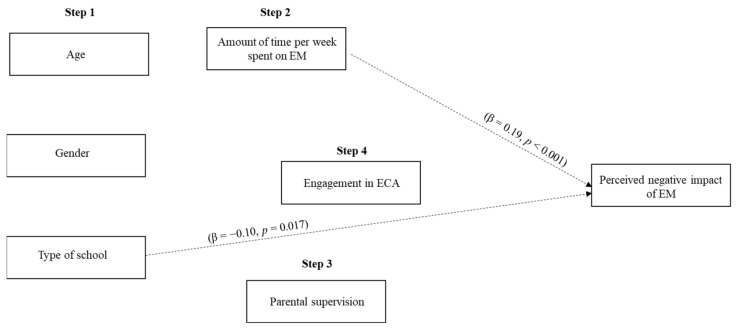
Predictors of perceived negative impact of EM (parents’ sample).

The same hierarchical regression was conducted for parents (*F*(6,543) = 6.42, *p* < 0.001, *R^2^* = 0.07 (see Figure 2)). Only type of school (*b* = −0.10, β = −0.10, *t* = −2.39, *p* = 0.017) and time they think their children spend per week on EM (*b* = 10.64, β = 0.19, *t* = 4.40, *p* < 0.001) were predictors of parents’ perceived negative impact of EM on their children. Parents whose children went to public schools and who perceived their children as spending more time on EM perceived a higher negative impact of these devices on their children. Children’s age (*p* = 0.753), sex (*p* = 0.339), the existence of rules (*p* = 0.118), and engagement in ECAs (*p* = 0.274) were not significant predictors.

## 4. Discussion

We aimed to explore the protective role of participation in ECAs and of parental mediation in the use of EM by youths. Consistent with previous literature (e.g., [11]), EM use and its negative impact were positively associated with youths’ ages. The results also showed that the youth sample reported spending longer on EM than their parents perceived, as found in previous research [41], although no statistically significant differences were found regarding the perceived negative impact of EM use reported by both groups. Additionally, when perceptions related to the time spent on EM were similar (youth–parent congruency), the perceived negative impact of EM use was lower for both youth and their parents.

EM use, and particularly screen media use, is a concerning trend, and little is known about the congruence between parents and children regarding its use by children [42]. The differences found between youths and parents regarding the use of EM may have two main explanations: the possible different meanings of the “use of EM”, and other concepts related; and the parents’ ability to accurately evaluate the use of EM.

The first possible explanation for the disagreement stems from the ubiquity of EM in our daily lives, especially for a generation that was born in the digital era. This ubiquity (arising for example from the use of mobile screens), as well as multitasking (which seems to be a routine in children of all ages), may lead to different meanings of the “use of EM” and, consequently, determining a different assessment of this use. Other concepts, such as what constitute EM devices, can also be understood differently by parents and children, and contribute to the disagreement found. For most parents, watching TV probably occurs on an actual television set, while for youths it can be on a TV, a smartphone or a computer. Thus, youths and parents may have different perceptions about what to report regarding EM use, and what are some devices objectively included in the EM concept. In future studies, it will be important to ensure that all participants have the same interpretations of the basic concepts to be assessed, such as “use of EM”, or devices such as “TV”. In addition to these methodological issues, the results also suggest the necessity of a focus on the assessment of the quality of parental mediation and its relation to the congruence vs. incongruence or discordance between informants.

The disagreement can also be explained by the parents’ ability to accurately evaluate the use of EM by their children, which seems to be dependent on the degree of autonomy of the latter in that use. As Wood et al. [42] suggest, younger children are more dependent on their parents for the use of EM and, therefore, parents’ reports of use may be relatively accurate. Regarding older children and adolescents, parents’ determination of the time spent using EM may be less accurate due to less parental supervision. In this study, the sample was composed of adolescents and, as such, the determination of the time of use of EM by the parents may have been less accurate.

The result related to the congruence associated with a lower perceived negative impact of EM use may reveal a pattern of trusting communication and openness between parents and youths which, in turn, could function as a protective factor in relation to this use. These results can be understood in the context of studies that highlighted the role of family relationships in this domain. For example, Kerr et al. [35] found that parents’ knowledge of their children’s daily activities was more related to what children shared within the family and less with monitoring efforts from the parents. Similar results were found by researchers focusing on EM use, wherein family environments characterized by trust and closeness showed an increased likelihood of youths reporting worrying situations related to the use of EM [16].

Most participants reported that their parents defined rules related to the use of EM. These participants reported spending less time on EM compared to youth who reported no rules, which is consistent with parents’ perceptions. However, perceptions of the negative impact did not differ based on the definition of rules, which is consistent with the literature [30]. Parental restrictions are often related to rules about time spent on these devices, but not necessarily to the management and the openness of talking about what concerns them online. As mentioned, the possible quality of communication between children and parents that may exist in this sample seems to be an intra-family protective factor in the use of EM. One limitation of this study was the assessment of parental mediation, using a dichotomous question regarding rules or restrictions in the use of EM. It would be interesting for future studies to further explore different types of mediation strategies and their impact on the use of EM, as well as their perceived negative impact. This could provide a more in-depth assessment of parental mediation using instruments, such as the recently published scale of parental mediation of social media by Ho et al. [43].

Most young people reported being involved in some type of ECA, an expected feature of this sample, since this engagement is described as a common developmental experience for many children and youth [8,25]. Those who engaged in ECAs reported spending less time on EM and perceived less of a negative impact of these devices, which is consistent with their parents’ perceptions. These results are in line with previous literature suggesting that young people who participate in ECAs have part of their after-school time allocated to these activities and, therefore, do not spend as much time on EM. As ECAs are considered an aspect of positive development and an enabler of social and emotional skills, extended over time and in different contexts [44,45], the youths engaging in ECAs may develop a larger set of skills that allow them to be more protected from the negative impact of EM use. Besides the socioemotional skills, the promotion of the time structure provided by ECAs can contribute as an important protective factor external to the family context. Future research should also focus on the type (e.g., sports vs. music), breadth, intensity, duration, regularity, and motivation to participate in ECAs, as well as other factors that could influence youth’s use of EM and ECAs (e.g., socioeconomic status).

When analyzing the predictive power of ECA engagement, regardless of time spent per week on EM, engaging in ECAs was a significant predictor of perceiving less of a negative impact of EM use for both youth and parents, confirming the role of ECA as a protective factor. These results reinforce what has been discussed previously: ECAs are not just activities that allow young people to occupy their time—they contribute to lessening the use of EM. The engagement in ECAs is a protective factor in the use of EM by young people, not because of reducing the time available to use EM, but probably due to the socioemotional skills that these activities allow them to develop, making them more competent in this use and having fewer negative experiences. Previous research [26,45] demonstrated the benefits of ECA engagement for socioemotional wellbeing. Youth who have the opportunity to develop, through ECAs, for example, a better self-concept and self-esteem, and who learn to better self-regulate their emotions, will probably be more able to cope with the challenges posed by EM use, regardless of the amount of time spent on EM.

Parents’ definition of rules was only a marginally significant predictor of the negative impact of EM use. This result confirms the idea that the definition of rules does not by itself constitute a protective factor, particularly in the age group of this sample, as adolescents require different levels of parental supervision, being more dependent on the quality of parent–child communication [46]. Notably, in the parents’ model, the main predictor of the perception of a less negative impact of the use of EM by their children was the amount of time their children spent on EM devices, which seems to lead to the same idea: the less the use, the lesser the negative impact.

Finally, these differences in the predictive models of young people and parents are relevant in the current context, especially in a post-COVID-19 era in which this greater use of EM is expected to continue. Therefore, the time of use of EM seems to be increasingly intangibly, and its lesser use does not seem to play a protective role for young people. What protects them are the relationships and the skills they develop in the context of face-to-face interaction, which is a positive sign.

## 5. Conclusions

We tested the role of ECA engagement and parental mediation on the impact of the use of EM by young people. Parental mediation was examined through two indirect variables: the existence of rules defined by parents regarding youth EM use, and the congruency between parents’ and youth’s assessment of time spent by youth with EM. Regardless of time spent per week with EM, engaging in ECAs was a significant predictor of perceiving less of a negative impact of EM, while parents’ definition of rules was only a marginally significant predictor. However, the results showed that parents and youth who are congruent in terms of the assessment of time spent by youth in EM tend to perceive a less negative impact of EM use.

The data in this study were collected in a pre-pandemic COVID-19 period, which reinforces the practical usefulness of the present study, since EM has become the current background of young people. Thus, more than focusing on limiting the time they are connected to EM, it is crucial to involve youth in activities during which they may develop socioemotional skills, empowering them to deal with the risks of using EM. On a socio-political level, our findings highlight the need to provide inclusive access to ECA, particularly for disadvantaged populations. Furthermore, educational strategies related to this subject should prioritize parental education, focusing on the relevance of ECA engagement. It is important to provide information on the positive impact of ECAs for children’s development, which is key for parents’ understanding of the need to create conditions for children to regularly engage in ECAs, and to maintain their interest in these activities. Moreover, the professionals responsible for these activities could benefit from training opportunities aimed at developing socioemotional skills in young people (e.g., self-esteem, self-confidence, problem-solving skills, seeking or offering support and help when needed, emotional control), which may buffer the potential negative impact of the use of EM. In addition, the results from this study reinforce the need to help parents replace the use of predictable restrictive mediation via more effective communication-based mediation regarding EM use.

## Figures and Tables

**Table 1:** Youths’ sociodemographic characteristics.

Sociodemographic Variables	*n*	Percentage
**Sex**		
Female	414	57%
Male	313	43%
Did not report	2	
**Grade**		
7th	205	28%
8th	333	46%
9th	191	26%
**School**		
Public	266	36%
Private	371	51%
Did not report	92	13%
**School region**		
North	98	13%
Center	147	20%
Lisbon	57	8%
Alentejo	57	8%
Algarve	31	4%
Azores	26	4%
Madeira	221	30%
Did not report	92	13%

**Table 2:** Frequency and characteristics of the groups formed based on the difference reported by parents and children regarding the time per week children spent on electronic media (EM).

Group	*n* (%)	Reported Time Range
Parents > Children (discrepancy)	206 (33%)	−9 h 40 min to −1 h 21 min
Parents = Children (congruency)	205 (33%)	−1 h 20 min to 0 h 37 min
Parents < Children (discrepancy)	208 (34%)	0 h 38 min to 31 h 08 min

**Table 3:** Means, standard deviations, and correlations between age, time spent with electronic media (EM), time spent on extracurricular activities (ECAs), and perceived negative impact of EM.

Variables	1.	2.	3.	4.	5.	6.
1. Age	13.46 (1.03)					
2. Time per Week Spent on EM (Youth)	0.26 ***	9 h 31 min (6 h 38 min)				
3. Perceived Negative Impact of EM (Youth)	0.14 ***	0.26 ***	0.69 (0.39)			
4. Time per Week Spent on ECA (Youth)	0.07	0.04	–0.12 **	4 h 23 min (2 h 88 min)		
5. Time per Week Spent on EM (Parents)	0.25 ***	0.57 ***	0.19 ***	–0.10 *	8 h 59 min (7 h 28 min)	
6. Perceived Negative Impact of EM (Parents)	0.10 **	0.23 ***	0.47 ***	–0.17 ***	0.25 ***	0.72 (0.48)

* *p* < 0.050, ** *p* < 0.010, *** *p* < 0.00.

## Data Availability

The data presented in this study are available on request from the corresponding author.
